# Decoding the Secret of Cancer by Means of Extracellular Vesicles

**DOI:** 10.3390/jcm5020022

**Published:** 2016-02-04

**Authors:** Nobuyoshi Kosaka

**Affiliations:** 1 Division of Molecular and Cellular Medicine, National Cancer Center Research Institute, 5-1-1, Tsukiji, Chuo-ku, Tokyo 104-0045, Japan; nkosaka@ncc.go.jp; Tel.: +81-3-3542-2511 (ext. 4809); 2 Department of Zoology, University of Oxford, Tinbergen Building, South Parks Road, Oxford OX1 3PS, UK; 3 JSPS Postdoctoral Fellow for Research Abroad, 5-3-1, Kojimachi, Chiyoda-ku, Tokyo 102-0083, Japan

**Keywords:** extracellular vesicles, exosomes, microRNA, microenvironment cell, metastasis, dormancy, cancer initiation, recurrence, brain metastasis, biomarker

## Abstract

One of the recent outstanding developments in cancer biology is the emergence of extracellular vesicles (EVs). EVs, which are small membrane vesicles that contain proteins, mRNAs, long non-coding RNAs, and microRNAs (miRNAs), are secreted by a variety of cells and have been revealed to play an important role in intercellular communications. These molecules function in the recipient cells; this has brought new insight into cell-cell communication. Recent reports have shown that EVs contribute to cancer cell development, including tumor initiation, angiogenesis, immune surveillance, drug resistance, invasion, metastasis, maintenance of cancer stem cells, and EMT phenotype. In this review, I will summarize recent studies on EV-mediated miRNA transfer in cancer biology. Furthermore, I will also highlight the possibility of novel diagnostics and therapy using miRNAs in EVs against cancer.

## 1. Introduction

Dr. Jan Lotvall’s group was the first to discover microRNA (miRNA) transfer [1]. In their 2007 paper, the authors showed the transfer of variable RNA, such as mRNA, long non-coding RNA (lncRNA), and microRNA (miRNA), between cells through extracellular vesicles (EVs). EVs are small membranous vesicles that are secreted from numerous types of cells and function in intercellular communication by transporting intracellular contents, such as protein and RNA [2]. EVs, including exosomes, microvesicles, and other types of membrane vesicles found in various body fluids such as blood, urine, and saliva, are differentiated by their mechanisms of biogenesis and secretion [3]. miRNA, which is the small RNA molecule inhibiting the gene function by interacting with the 3′UTR of those genes [4,5], had been thought to function only inside the cells that expressed those miRNAs. After this, three papers, including ours, first showed the function of transferred miRNAs in the recipient cells, such as immune cells [6], cancer cells [7], or endothelial cells [8]. These papers opened up a novel research field in which miRNAs may serve as novel humoral factors in cell–cell communication. Indeed, the current focus in this field is on the roles of exosomal miRNA between cancer cells and microenvironmental cells in cancer development [9].

**Figure 1:**
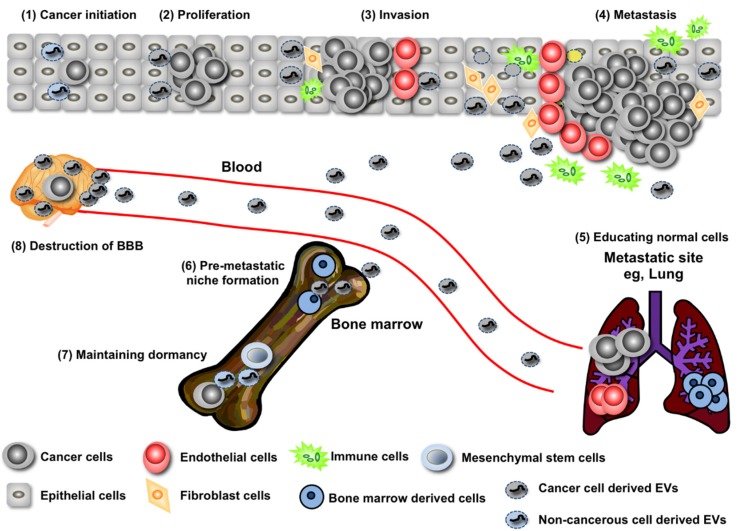
EVs from cancer cells manipulate the cells in their microenvironment. EVs are involved in every step of cancer development. In cancer’s initiation stage, normal cells prevent the outgrowth of cancer cells by secreting tumor suppressive miRNAs through EVs (1); however, the cancer cells can avoid this inhibitory machinery, finally resulting in a tumor expansion (2). Cancer cells exhibit horizontal transfer of genes that promote proliferation by EVs from cancer cells harboring those genes to cancer cells that do not harbor those genes (2); Many reports have shown that cancer cell-derived EVs promote cancer malignancy (3,4). In addition, cancer cell-derived EVs activate fibroblasts, leading to extracellular matrix degradation and the induction of cancer-promoting cytokines (3,4). When the tumor microenvironment is hypoxic, cancer cells secrete angiogenesis-inducing EVs that help to overcome oxygen and nutrition deficiency by activating endothelial cells to form the vascular system (3,4). These will contribute to further cancer development, such as metastasis (4); EVs derived from cancer cells infiltrate bone marrow cells, leading to the formation of a pre-metastatic niche that is prepared by bone marrow cells (5); In addition, EVs from cancer cells directly affect the metastatic site to induce angiogenesis (6). Transfer of miRNAs by EVs from the bone marrow mesenchymal stem cells regulate breast cancer cell dormancy in a metastatic niche (7). Furthermore, mechanism of brain metastasis mediated by EVs triggers the destruction of BBB (8).

In this review, I will summarize current knowledge regarding the contribution of miRNAs in EVs during cancer development, such as initiation, invasion, metastasis, and recurrence. Furthermore, I will discuss therapeutic approaches using EVs and miRNAs, which are originally from cancer cells and/or microenvironmental cells, in EVs for diagnosis and treatment of cancer (Figure 1).

## 2. EV miRNA’s Contribution to Both the Promotion and Suppression of Cancer Initiation

Numerous studies have shown a broad variety of mechanisms for tumor initiation, including gene amplification/deletion/mutation, cellular stress, metabolic alteration, and epigenetic changes [10]. In addition to those cell-autonomous mechanisms, non-cell-autonomous mechanisms also contribute to cancer initiation. Recent research has shown that the EVs from noncancerous neighboring epithelial cells have the capacity to suppress the expansion of cancer initiation [11]. It has been shown that one tumor-suppressive miRNA, miR-143, whose expression in normal prostate cell lines is higher than that in prostate cancer cell lines [12], transfers growth-inhibitory signals to cancerous cells *in vitro* and *in vivo* in EVs released from noncancerous cells. During cancer initiation, it has been shown that there are some fights between newly emerged cancerous cells and the surrounding epithelial cells [13,14]. Taken together with these results and publications, I hypothesized that growth inhibitory miRNAs are actively released from noncancerous cells to suppress the growth of abnormal cells with a partial oncogenic ability, thereby restoring them to a healthy state [11]. Because abundantly existing healthy cells continuously provide nascent overproliferative cells with tumor-suppressive miRNAs for a long period, it can be assumed that a local concentration of secretory miRNAs can become high enough to restrain tumor initiation. Indeed, employing the copy number analysis of miRNA in EVs, it has been proposed that the large numbers of EVs produced per cell allows for the loading of low miRNA numbers per EVs to achieve functional relevance [15]. In many cases, the expression of tumor-suppressive miRNAs is downregulated in cancer cells [16]; therefore, this continuous provision of tumor-suppressive miRNA through the EVs could be a homeostatic mechanism that tumor cells need to overcome. Although further studies are essential to clarify this hypothesis, understanding the preventing mechanisms of cancer initiation by surrounding cells might be important to realize the prevention of cancer.

Furthermore, this biological process has been confirmed between other combinations of cells, such as multiple myeloma and bone marrow mesenchymal stromal cells (BM-MSCs) [17]. In this case, EVs isolated from BM-MSCs of patients with multiple myeloma induced multiple myeloma tumor growth *in vivo* and promoted dissemination of tumor cells to the BM in an *in vivo* translational model of multiple myeloma. Moreover, the levels of miR-15a were significantly higher in EVs from normal BM-MSC-derived EVs than those in EVs from multiple myeloma BM-MSCs, suggesting the tumor-suppressive role of MSC-derived miR-15a from EVs against multiple myeloma. It has been known that miR-15a acts as a tumor-suppressive miRNA and that multiple myeloma growth can also be suppressed by miR-15a [18,19]. As in the relationship between prostate epithelial cells and prostate cancer cells shown above [11], BM-MSCs seem to guard against cancer cells by providing tumor-suppressive miR-15a against multiple myeloma; however, for some reason, the expression of miR-15a is downregulated in BM-MSCs, and the EVs from those BM-MSCs are no longer able to suppress the expansion of multiple myeloma. One of the main reasons for downregulation of tumor-suppressive miRNAs, such as miR-143 and miR-15a, is deletion from the genome [18]; however, this deletion has been reported in cancer cells. Thus, other downregulation mechanisms of tumor-suppressive miRNA should exist in those normal cells. As shown above, the secretion of miR-143 or miR-15a in EVs from noncancerous cells is important for suppressing cancer initiation; revealing the regulatory mechanisms of miRNAs in noncancerous cells might also lead to answers regarding the mechanisms of cancer initiation.

Although a variety of noncancerous cells surround cancer cells, because of the low secretion level of EVs from noncancerous cells, a single species of miRNA in EVs is insufficient for suppressing the expansion of cancer cells. However, considering that EVs carry a variety of molecules, not only the many tumor-suppressive miRNAs but also another protein with anti-cancer activity that does exist in EVs might contribute greatly to the suppression of cancer cell expansion by surrounding noncancerous cells. In EVs, for instance, PTEN, one of the most commonly lost tumor suppressors in human cancer, can transfer into other cells and reduce phosphorylation of the serine and threonine kinase Akt, resulting in the reduction of cellular proliferation in recipient cells [20]. The role of EV miRNAs in cancer initiation has not yet been clarified; however, considering the current incidence of cancer, how to prevent cancer is a question that must be answered.

## 3. Regulation of Cancer Progression by miRNAs in EVs

EVs from cancer cells affect other cancer cells in the heterogeneous population of a tumor, resulting in the transfer of metastatic capability. Most cancer cells release a variety of EV types, and the transfer of EVs dictates the behavior of the recipient cell for their benefit. Much of this research involved *in vitro* studies; however, the behavior of EVs *in vivo* needs to be addressed. Recently, significant work was published regarding the EV exchange between tumor cells by combining high-resolution intravital imaging with a Cre-LoxP system to trace the behavior of EVs *in vivo* [21]. Less malignant breast cancer cells located within the same and within distant tumors take up EVs secreted by highly metastatic breast cancer cell lines. These EVs carry mRNAs involved in migration and metastasis, resulting in the promotion of migratory behavior and metastatic capacity. For instance, the miR-200 family, which regulates the mesenchymal-to-epithelial transition, was secreted in EVs from metastatic breast cancer cell lines; this miR-200 transfer to the non-metastatic cancer cells altered gene expression and promoted mesenchymal-to-epithelial transition [22]. Drug resistance in neuroblastoma (NBL) is another example of the role of miRNA in EVs [23]. Co-culture experiments were performed that showed the transfer of miR-21 from NBL cells to human monocytes and miR-155 from human monocytes to NBL cells by EVs. miR-21 in EVs secreted from NBL cells bind to TLR8 in human monocytes, resulting the activation of NF-кB pathway activation in monocytes. On the other hands, miR-155 levels in human monocytes were progressively increased by the NBL-derived EVs and this led to the accumulation of miR-155 in EVs from human monocytes. This miR-155 suppresses the expression of TERF1, which is an inhibitor of telomerase, resulting in the promotion of chemoresistance in NBL [24]. These data indicate a unique role of miR-21 and miR-155 in the crosstalk between NBL cells and human monocytes in their resistance to chemotherapy. In addition to NBL, it has been shown that miRNAs in EVs contribute to the growth of HCC (hepatocellular carcinoma) [25]. In this report, the miRNAs that were highly expressed in EVs from HCC can modulate TAK1 expression, resulting in the enhancement of transformed cell growth in recipient cells.

It is already clear that microenvironmental cells contribute greatly to cancer development, which enhances the cancer cell’s capacity to metastasize to other organs [10]. The microenvironment contains many factors and cell types, such as immune cells, fibroblasts, and endothelial cells, which influence cancer progression. The secretion of humoral factors from microenvironmental cells to cancer cells is essential for metastasis during cancer development.

Endothelial cells contribute to vascular generation and provide cancer cells with oxygen and nutrition, which are difficult to obtain in a tumor [26]. In this case, cytokines, such as VEGF or bFGF, are the molecules responsible for communication between cancer cells and endothelial cells. Recently, it has been shown that EVs from cancer cells contain various molecules that promote angiogenic cytokines, such as VEGF, bFGF, and TGF-beta. In addition to these angiogenic cytokines, there are multiple secretory miRNAs in EVs that have been reported to promote angiogenesis. For instance, miR-9 in EVs from cancer cells educed SOCS5 levels, leading to the activated JAK-STAT pathway, resulting in the promotion of endothelial cell migration and tumor angiogenesis [27]. In addition, miR-210, which was known to act as angiogenic miRNA, transfers from cancer cells to the endothelial cells through the EVs and promotes angiogenesis under the regulation of nSMase2 [28], which has known to regulate EV production [7,29]. In endothelial cells, the expression of Ephrin A, the target gene of miR-210 [30], was downregulated after the transfer of EVs from cancer cells. In addition, *in vivo* studies showed the effect of miRNA transfers from cancer cells. Indeed, manipulating the expression of nSMase2 can affect EV production, and this manipulation affects the metastatic ability as well. Indeed, miR-210 has been known to regulate angiogenesis in endothelial cells [30] and iron homeostasis in cancer cells [31] and was upregulated by a hypoxic condition [30].

## 4. Long-Distance Regulation of Metastasis by miRNAs in EVs

EVs affect not only cells close to cancer cells but also cells in distant tissues. For instance, highly metastatic melanoma-derived EVs increased the metastasis of primary tumors by educating the bone marrow [32]. In addition, miR-122 in EVs from cancer cells downregulated the glycolytic enzyme pyruvate kinase in non-tumor cells located in the pre-metastatic niche, resulting in the suppression of glucose uptake by non-tumor cells in the pre-metastatic niche and increased nutrient availability to cancer cells in the pre-metastatic niche [33].

EVs seem to tightly associate with brain metastasis, an important cause of mortality in cancer patients. Brain metastasis is associated with a particularly poor prognosis for cancer patients; however, the detailed molecular mechanisms have not yet been clarified. One of the key features of brain metastasis is the destruction of the blood–brain barrier (BBB) and the migration of cancer cells through the BBB [34,35], which consists of the endothelium and surrounding cells including pericytes and astrocytes [36,37] and limits the passive diffusion of molecules. Tumor cells recognize and bind to components of the vascular membrane, thereby initiating extravasation, invasion of cancer cells through the BBB, and the beginning of new growth at secondary organ sites [38,39]. Variable potential molecules promote or disturb BBB destruction. The contribution of EVs in brain metastasis was also reported. EVs from brain metastatic cancer cells contained miR-181c, and it transferred to brain endothelial cells, resulting in the destruction of tight junction proteins of BBB, such as Claudin-5, Occludin, and ZO-1 [40]. The primary cytoskeletal protein, actin, has been known to bind all ZO proteins, claudin, and occludin. Phosphorylation of cofilin through 3-phosphoinositide-dependent protein kinase-1 (PDPK1), which is the target gene of miR-181c, is thought to inactivate cofilin in a spatial manner, in which local activation occurs in the cell membrane [40]. In addition, tight junction proteins are also the direct targets of miRNA in EVs. Indeed, miR-105 in EVs from breast cancer cells suppressed ZO-1 expression in endothelial cells, resulting in the loss of cell–cell adhesion and leading to the promotion of metastasis [41].

These reports suggest that miRNAs in EVs from brain metastatic cancer cells targeted tight junction proteins and regulators of actin proteins simultaneously, resulting in the efficient destruction of the BBB for metastasis.

## 5. Recurrence Rebooted by EVs

Recent successful early detection and effective systemic adjuvant therapy has caused a decrease in mortality from breast cancer. However, breast cancer often recurs, typically within five years but even up to 10 to 20 years after surgery [42]. In addition, breast cancer recurrence is often more aggressive and untreatable. The usual explanation is that breast cancer cells survive for a long time somewhere in the body in a state of cancer dormancy. Dormant cancer cells cease dividing but survive in a quiescent state while waiting for appropriate environmental conditions to resume proliferation. It has been shown that breast cancer cells can be detected in the bone marrow (BM) in early stages of breast cancer [43]. It is thought that micrometastases form in the bone marrow and then recirculate to invade other, distant organs [44], thus understanding the molecular mechanisms for keeping cancer cells dormant by interacting with their microenvironmental cells is essential for diagnosing and preventing the recurrence of cancer. However, little is understood about this molecular mechanism. Recently, it was revealed that BM-MSCs play an important role in inducing dormancy in breast cancer cells in bone marrow through the transfer of a cell cycle inhibitory miRNA by EVs [45]. In this situation, miR-23b in EVs from BM-MSCs promoted dormancy through the downregulation of MARCKS, which is a target gene of miR-23b in breast cancer cells. Therefore, transfer of miR-23b by EVs and its suppression of MARCKS, one of the mechanisms for cancer recurrence, result in the suppression of the cell cycle and the transition to dormancy in breast cancer cells.

It is tempting to postulate the possibility of answering the following questions: (1) What is the physiological effect of miR-23b in EVs? (2) What is the molecular mechanism for avoiding dormancy of breast cancer cells? Answering these questions will lead to predicting the existence of breast cancer cells in bone marrow, or allow us to make drugs that target breast cancer cells in the bone marrow.

## 6. EVs as a New Diagnostic Tool

It has been shown that EVs reflect the physiological state of their cells of origin [46], and almost all types of cells, including cancer cells, secrete EVs that contain specific proteins and miRNAs into their microenvironment and circulation [3,47]. Because of this, EVs can be found in various body fluids, such as blood, urine, and saliva [2,48]. Taking these facts into account, EVs provide a rich source of potential biomarkers.

As shown above, the first indication regarding circulating RNA as a biomarker was the discovery of mRNA and miRNA in EVs [1]. After this, it was shown that miR-21 in EVs enriched in the serum of glioblastoma patients was expressed at higher levels in the serum of patients compared with normal controls [49]. This report became an impetus for research into biomarkers that use exosomal miRNAs in various types of cancer, and a specific miRNA profile has been published [3,50]. Moreover, miRNAs can be readily detected in small sample volumes using specific and sensitive quantitative real-time PCR (qRT-PCR) [51]. Many studies have evaluated the feasibility of circulating miRNAs for detecting cancer for diagnosis and for prognostic/predictive markers [49,52,53,54,55,56,57,58,59,60,61]. For instance, the amount of circulating miR-210 is significantly higher in serum from circulating tumor cell (CTC)-positive metastatic breast cancer patients compared with that in plasma from CTC-negative metastatic breast cancer patients and controls [62]. In addition, circulating miR-210 levels were significantly higher in individuals with residual disease than in those who achieved a pathologically complete response to trastuzumab [63], suggesting that circulating miR-210 can be used to predict and perhaps monitor responses to therapies involving the use of trastuzumab. As shown above, EVs isolated from metastatic breast cancer cells promote metastasis via the induction of angiogenesis in the tumor [28]. These EVs contain multiple miRNAs that promote angiogenesis by regulating the gene expressed in endothelial cells, and it has been shown that miR-210 was included in this type of miRNAs [30]. Furthermore, as shown above, circulating miR-181c [40] and miR-105 [41] can be found in the serum from brain metastatic cancer patients. These data give hope for the treatment of brain metastasis, as the detection of circulating miRNA, such as miR-181c or miR-105, leads to the finding of brain metastasis.

This kind of approach against sera from patients with cancers, such as prostate cancer, colorectal cancer, and pancreatic cancer, has been widely investigated recently. Although there are still some issues to be resolved in using miRNA in EVs from serum, such as isolation methods, inner control, and detection methods, miRNA in EVs from serum/plasma will be a great help in the diagnosis of cancer or the monitoring of cancer during treatment. Combined with other cancer biomarkers that have already been used, this might be the earliest method for clinical usage of miRNAs in EVs as a cancer biomarker.

## 7. EVs Are Novel Therapeutic Target in Cancer

**Figure 2:**
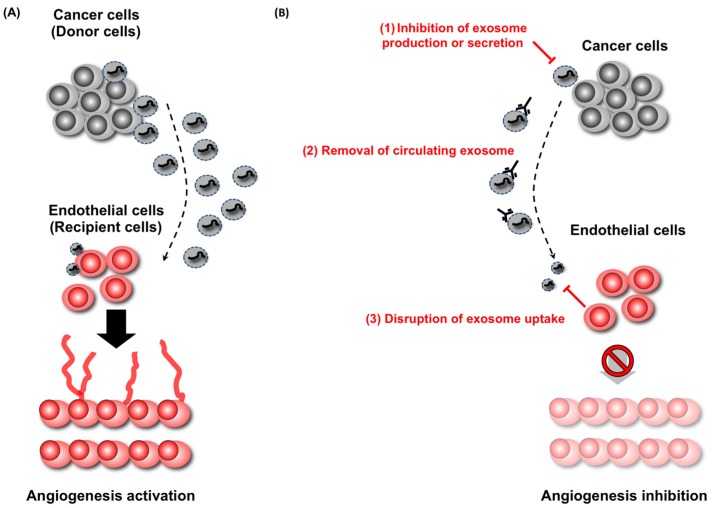
Therapeutic strategies against cancer-derived EVs. EVs are secreted from cancer cells and delivered to recipient cells, modulating the phenotype of the recipient cells. For instance, EVs from cancer cells are delivered to endothelial cells, which enhance angiogenesis (**A**). In this case, there are three therapeutic applications (**B**): (1) inhibition of EVs production from cancer cells; (2) elimination of circulating EVs from cancer cells; and disruption of EVs uptake by recipient cells (3). These therapeutic applications will prevent the delivery of EVs from cancer cells to endothelial cells, leading to the suppression of development of cancer cells.

The great contribution of EVs and miRNAs during cancer development has been shown, and their utilization for diagnosing or monitoring cancer is now a promising method for preemptive or personalized medicine. In addition to the diagnostic usage of EVs, targeting EVs for cancer treatment is becoming realistic as well (Figure 2).

Targeting molecules secreted through EVs from metastatic niches may prevent or delay cancer recurrence. In this situation, targeting the molecules related to EV secretion and/or production will be good candidates for EV-targeting treatment, such as nSMase2 [22,28,64,65], RAB27A, RAB27B [32,66,67], and RAB22A [68]. The suppression of these molecules leads to the inhibition of EV production, which results in the disruption of cancer development; however, it is essential to understand the contribution of these molecules in the secretion of EVs from not only cancer cells but also normal cells. Otherwise, disturbing the secretion of EVs from normal cells might influence the homeostatic function of EVs as shown above.

Another method for targeting EVs for cancer treatment involves capturing the circulating EVs from cancer cells. As shown above, cancer cells secrete some EVs from outside of the original tumor position. Thus, the complete elimination of circulating EVs will be a great benefit to cancer patients. For instance, EVs with human epidermal growth factor receptor type 2 (HER2) isolated from HER2 over-express breast cancer cells; this interferes with the activity of the therapeutic antibody Herceptin in the breast cancer patient. From this point of view, a device that could eliminate target EVs from the entire circulatory system would be a great benefit in cancer treatment. Indeed, this type of device has already been proposed and developed [69]. This technology immobilizes exosome-binding lectins and antibodies in the outer-capillary space of plasma filtration membranes that integrate into existing kidney dialysis systems.

Another possible therapeutic approach against EVs is to disturb EV absorption in recipient cells. Based on current reports, there are some tropisms for receiving EVs. For example, EVs from brain metastatic breast cancer cells tend to incorporate into endothelial cells but not into astrocytes or pericytes [40]. Only a few reports deal with the recipient mechanisms for EVs; however, understanding this process will give us another way to disturb the progression of cancer.

As shown above, tumor-suppressive miRNAs can attenuate the growth of cancer cell proliferation. Indeed, miR-143-tansduced EVs can suppress the proliferation of cancer cells *in vivo* [9]. Thus, it is tempting to postulate that this tumor-suppressive miRNA and EV combination might be useful for cancer treatment. In addition, EVs can efficiently deliver another tumor-suppressive miRNA, let-7, to epidermal growth factor receptor (EGFR)-expressing breast cancer cells [70]. Targeting has been achieved by engineering the donor cells to express the transmembrane domain of platelet-derived growth factor receptor fused to the GE11 peptide. These reports suggest that EVs can be a vehicle for tumor-suppressive miRNAs to attack cancer cells in patients.

## 8. Conclusions

Unlike the first discovery of exosomes in the 1980s [71], which thought of exosomes as the garbage of the cells, current research indicates that EVs including exosomes might be central mediators of cell–cell communication. Much research has been done in the past several years; however, we need to continue so that we can understand EV function and character in more detail. Then, we will be able to use EVs freely in clinical situations.
